# Immunoglobulin G and neutralizing antibody response after CoronaVac

**DOI:** 10.1590/1806-9282.20250360

**Published:** 2026-01-09

**Authors:** Pedro Borges Carvalho de Assis, Luca Nascimento Ferreira, Fernanda Medeiros Vale Magalhães, Paula Fernandes Távora, Luara Isabela dos Santos

**Affiliations:** 1 Faculdade Ciências Médicas de Minas Gerais – Belo Horizonte (MG), Brazil.; 2 Universidade Federal de Minas Gerais – Belo Horizonte (MG), Brazil.

**Keywords:** COVID-19 vaccines, Neutralizing antibodies, SARS-CoV-2, Antibodies, Immunoglobulin G, Neutralization tests

## Abstract

**OBJECTIVE:**

The aim of this study was to evaluate the kinetics of immunoglobulin G and neutralizing antibody responses following vaccination with CoronaVac in healthcare professionals.

**METHODS:**

A cohort of 35 healthcare professionals was recruited, and blood samples were collected to assess immunoglobulin G detection and neutralizing antibodies against severe acute respiratory syndrome coronavirus 2.

**RESULTS:**

Immunoglobulin G seroconversion peaked at D30 (68.2%, 15/22), followed by stabilization at D48 and D90. Logistic regression confirmed a significant association between time post-vaccination and seroconversion (p=0.0043). Neutralizing antibody analysis showed a stable detection rate for Nab V1 (Wuhan and B.1.1.7) between D48 and D90 (p>0.9999), while Nab V2 (B.1.351 and P.1) remained consistently lower. Among immunoglobulin G-positive individuals, 92.3% (12/13) had neutralizing antibodies at D48, decreasing to 42.8% (6/14) at D90.

**CONCLUSION:**

CoronaVac effectively induces immunoglobulin G seroconversion, peaking at D30. However, neutralizing responses to emerging variants were lower, suggesting the need for booster doses to maintain immunity. These findings highlight the importance of monitoring vaccine-induced immunity over time.

## INTRODUCTION

In March 2020, the World Health Organization (WHO) declared the severe acute respiratory syndrome coronavirus 2 (SARS-CoV-2) outbreak a pandemic, which has infected hundreds of millions of people with coronavirus disease 2019 (COVID-19)^1^. The rapid spread of the virus and its potential to cause severe acute respiratory syndrome led to a substantial increase in hospitalizations and fatalities^2^. The severity of this condition is primarily linked to the virus's ability to invade lung cells via binding to angiotensin-converting enzyme 2 (ACE2) receptors, with the Spike protein playing a central role in this process^3^.

Given the significant burden of severe cases, early ­therapeutic strategies included the administration of neutralizing ­antibodies to prevent viral entry into host cells^4,5^. These antibodies, whether derived from convalescent plasma or monoclonal ­therapies, demonstrated efficacy in reducing viral replication and ­mitigating the excessive inflammatory responses associated with severe COVID-19^6^. Neutralizing antibodies, particularly anti-S antibodies, specifically bind to the receptor-binding domain (RBD) subunit of the Spike protein, blocking its interaction with the ACE2 receptor and thereby preventing cellular infection^7^. Although the presence of anti-Spike immunoglobulin G (IgG) antibodies has been associated with improved clinical outcomes, it is important to note that not all IgG antibodies can directly inhibit viral entry^8^. Therefore, evaluating both total IgG levels and neutralizing antibody activity provides a more comprehensive understanding of vaccine-induced immune protection. In parallel with these therapeutic approaches, large-scale vaccination efforts were initiated to prevent SARS-CoV-2 infection and control viral spread. The first vaccine administered in Brazil was CoronaVac, developed by Sinovac and Butantan Institute. This vaccine, based on inactivated virus technology, was widely distributed across the country and played a crucial role in the initial phase of the national immunization effort^9^. Other vaccine platforms, such as messenger RNA (mRNA) vaccines and non-replicating viral vector vaccines, were also deployed. Despite their different mechanisms of action, all were designed to elicit an immune response targeting the Spike protein. In the early stages of the pandemic, vaccine effectiveness was primarily evaluated through reductions in severe ­disease and hospitalizations^10-12^.

Understanding the immune response induced by vaccination is essential for determining long-term protection and shaping future immunization strategies. Thus, this study aims to analyze the presence of IgG and neutralizing antibodies against SARS-CoV-2 following vaccination with CoronaVac. This vaccine was the first to be administered in Brazil and was broadly used during the early phase of the national immunization campaign. At that time, limited real-world data were available regarding the immunogenicity of inactivated virus vaccines. Therefore, focusing on this initial period provides important insight into the early performance of CoronaVac under large-scale public health implementation. In addition, it seeks to evaluate the kinetics and durability of the humoral immune response during the initial phase of Brazil's mass vaccination campaign.

## METHODOLOGY

### Study population

A total of 35 healthcare professionals were screened for the study in February 2021, during the initial phase of the vaccination campaign in Brazil. Volunteers were enrolled immediately before receiving the vaccination. All participants received two doses of the inactivated SARS-CoV-2 vaccine, CoronaVac (Sinovac Life Sciences Co., Ltd, Beijing, China), with a recommended interval of 28 days between doses. Informed consent was obtained from all participants, and the study was approved by the Ethics Committee of the Faculdade de Ciências Médicas de Minas Gerais (CAAE 40932620.9.0000.5134).

### Sample collection

Blood samples were collected at six time points: the day of vaccination and 10, 20, 30, 48, and 90 days post-vaccination (D0–D90). Capillary blood samples were used to perform the IgM/IgG assay at all time points, and peripheral blood samples were collected in serum-separating tubes on days 45 and 90 to perform the SARS-CoV-2 neutralization assay. These intervals were selected based on the known immunological kinetics of inactivated virus vaccines, which typically induce a peak antibody response within 2–4 weeks after the second dose. Day 30 was chosen to capture this peak IgG response, while Days 48 and 90 were included to assess short-term antibody persistence and early decline in both IgG and neutralizing antibodies. This schedule allowed for a more detailed evaluation of the early-phase immune response following vaccination with CoronaVac.

### Severe acute respiratory syndrome coronavirus 2 IgM/immunoglobulin G assay

IgM and IgG antibodies against SARS-CoV-2 were detected using the COVID-19 Ab Plus ECO test, a qualitative rapid immunochromatographic assay, following the manufacturer's instructions. This test has a reported sensitivity of 96% and specificity of 98%, according to the manufacturer (Anvisa MS 80954880160, TR.0087).

### Severe acute respiratory syndrome coronavirus 2 neutralization assay

Neutralizing antibodies were assessed using the ECO F COVID nAb fluorescence immunoassay, a qualitative assay performed according to the manufacturer's instructions. The test detects antibodies that inhibit the interaction between the RBD of the SARS-CoV-2 Spike protein and the human ACE2 receptor. Two types of neutralizing antibodies were assessed: Nab V1 (Wuhan, B.1.1.7) and Nab V2 (B.1.351 and P.1). The test device contains two lines: a control line coated with anti-RBD antibody and a test line coated with PEG-streptavidin. During the assay, europium-labeled RBD protein binds to biotinylated ACE2 to form a complex that competes with neutralizing antibodies in the sample. If neutralizing antibodies are present, they prevent the formation of this complex, leading to a reduction in fluorescence at the test line. The analyzer automatically calculates the inhibition rate and interprets the result as reactive (≥20% inhibition) or non-reactive (<20% inhibition). According to the manufacturer (Anvisa MS 80954880157), the test has a sensitivity greater than 99.9% and a specificity of 97.8%.

### Statistical analysis

Statistical analyses were conducted using GraphPad Prism. For the analysis of qualitative variables, Fisher's exact test was used. Normality was assessed using the Shapiro-Wilk test. The Kruskal-Wallis test was used to compare neutralizing antibody levels across time points, followed by Dunn's multiple comparisons test to identify specific differences between groups. Logistic regression was applied to evaluate the association between time post-vaccination and the probability of seroconversion, with results reported as odds ratios and 95%CI. A significance threshold of p<0.05 was considered.

## RESULTS

Initially, 35 volunteers consented to participate in the study. Of these, seven individuals were excluded due to incomplete sample collection (lost to follow-up). An additional four participants were excluded after testing positive for anti-SARS-CoV-2 IgG at baseline (D0) and reporting a prior COVID-19 diagnosis. Two participants contracted SARS-CoV-2 during the follow-up period and were due to intercurrent infection. As a result, 22 participants who completed all study visits and had no prior or intercurrent infection were included in the final analysis. The median age of participants was 30.0 years (interquartile range: 11.75), with the majority being female (72.7%, 16/22).

A progressive increase in reactivity of IgG anti-SARS-CoV-2 was observed over time, peaking at 68.2% (15/22) on D30, followed by stabilization on D48 and D90 (Table 1). A logistic regression model demonstrated a significant association between time post-vaccination and the likelihood of IgG seroconversion (β=0.0185, SE=0.0065, p=0.0043). Each additional day post-vaccination increased the odds of seroconversion by 1.87% (OR 1.0187, 95%CI 1.0058–1.0317).

**Table 1:** Immunoglobulin G seroconversion rates over time.

Days after the first dose	Non-reactive n (%)	Reactive n (%)
D0	22 (100%)	0 (0.0%)
D10	20 (91.0%)	2 (9.0%)
D20	19 (86.4%)	3 (13.6%)
D30	7 (31.8%)	15 (68.2%)
D48	9 (40.9%)	13 (59.1%)
D90	8 (36.4%)	14 (63.3%)

Seroconversion was defined as the presence of detectable immunoglobulin G antibodies.

**Figure 1:**
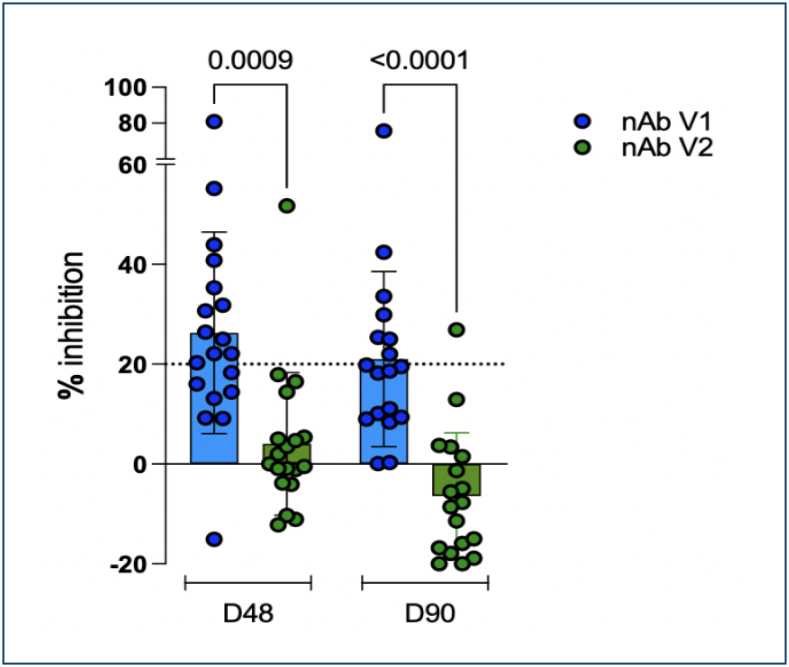
Percentage of inhibition for neutralizing antibodies (nAb) against severe acute respiratory syndrome coronavirus 2 variants at D48 and D90 post-vaccination with CoronaVac. The blue dots represent nAb V1 (targeting earlier variants), while the green dots represent nAb V2 (targeting more recent variants). The dotted line at 20% inhibition indicates the positivity threshold for neutralizing antibodies. Statistical significance was determined using the Kruskal-Wallis test followed by Dunn's multiple comparisons test (p=0.0009 for nAb V1 at D48 vs. D90; p<0.0001 for nAb V1 vs. nAb V2 at D90).

When evaluating neutralizing antibodies, results indicate a significant difference in the frequency of neutralizing antibody detection over time. Nab V1 remained relatively stable between D48 and D90 (p>0.9999), while Nab V2 showed a lower value in frequency between the two time points (p<0.0001) (Figure 1). It is important to note that the neutralizing antibody assay used in this study is qualitative. It provides binary results (reactive or non-reactive) based on a fixed inhibition threshold of ≥20%, without quantifying antibody titers. This limits the ability to assess gradual changes in neutralizing capacity over time.

At 48 days post-vaccination, among the 13 volunteers who tested positive for IgG using the chromatographic assay, 12 (92.3%) presented neutralizing antibodies against V1, while only one exhibited inhibition associated with the presence of Nab V2. At 90 days post-vaccination, among the 14 volunteers who tested positive for IgG via the chromatographic assay, 6 (42.8%) displayed Nab V1, and one individual who tested negative for IgG using the rapid test demonstrated the presence of neutralizing antibodies. These findings highlight the limitations of antibody detection using both methodologies, as well as the variability in individual immune responses.

## DISCUSSION

The results of this study demonstrate a progressive increase in IgG seroconversion following CoronaVac vaccination, peaking at D30 (68.2%, 15/22) and a stabilization during the period of the study. This pattern aligns with previous studies on inactivated virus vaccines, which report peak IgG responses ­approximately four to eight weeks post-vaccination^13^. The logistic regression model confirmed a significant association between post-vaccination time and the likelihood of IgG seroconversion (p=0.0043), indicating that the odds of seroconversion increased by 1.87% per day. These findings reinforce the effectiveness of CoronaVac in eliciting a robust humoral immune response.

However, a distinct kinetic pattern emerged when evaluating neutralizing antibodies. Nab V1 levels, which target early SARS-CoV-2 variants (Wuhan and B.1.1.7), remained relatively stable between D48 and D90 (p>0.9999). In contrast, Nab V2 ­levels, targeting later variants (B.1.351 and P.1), were consistently lower at both time points. It is important to note that the neutralizing antibody assay used in this study is qualitative, ­providing only a binary interpretation (reactive or non-reactive) based on a fixed inhibition cutoff (≥20%). As such, the assay does not yield quantitative titers, limiting the ability to detect gradual antibody declines or establish correlations with long-term clinical protection. This suggests a differential induction of ­neutralizing antibodies, with a lower response to variants of concern within the analyzed period. The persistently low Nab V2 levels are particularly concerning, as these variants have been associated with increased transmissibility and partial immune escape^14^.

Comparatively, mRNA vaccines have been shown to elicit higher neutralizing antibody titers that persist for longer ­periods. Goel et al. reported that, although neutralizing antibodies ­elicited by mRNA vaccines decline from peak levels, they remain stable for six to nine months post-vaccination^15^. In contrast, immunological modeling suggests that inactivated vaccines, such as CoronaVac, exhibit a more gradual and delayed maturation of neutralizing capacity, which may reflect a more rapid relative decline in early months^16^. Similar trends have been observed for other inactivated vaccines, including BBIBP-CorV (Sinopharm), which demonstrate lower and shorter-lived neutralizing antibody titers compared to mRNA vaccines^17^. Adenoviral vector vaccines, such as AstraZeneca (ChAdOx1-S) and Sputnik V, tend to generate a more sustained ­neutralizing response than inactivated vaccines, though still lower than that induced by mRNA platforms^18^. These variations highlight the importance of tailoring vaccine strategies according to the durability and breadth of protection.

Furthermore, several studies have shown that inactivated vaccines elicit weaker cross-neutralization against emerging variants. The CoronaVac-induced immune response has demonstrated reduced neutralization against Beta (B.1.351) and Gamma (P.1) variants, which may explain the persistently low Nab V2 levels in our study. This finding is consistent with reports indicating that inactivated vaccines generate robust responses against ancestral strains but struggle to neutralize later variants effectively^19^. The reduced ability to neutralize variants may be attributed to antigenic imprinting, where immune memory is more strongly retained for the original viral strain rather than newly emerging variants^20^.

Another important observation was the heterogeneity in immune responses among participants. While most individuals who seroconverted for IgG also developed neutralizing antibodies, some exhibited IgG positivity without detectable neutralizing antibodies, and vice versa. This discrepancy suggests that total IgG levels alone may not accurately predict neutralization capacity, underscoring the importance of functional assays in vaccine immunogenicity assessments^21^. Additionally, individual immune status and genetic background may influence the durability of vaccine-induced immunity. Cellular immunity and memory B-cell responses should also be considered as crucial components of long-term protection, as antibody levels alone may not fully represent immune competence^22^.

The implications of these findings extend to real-world vaccine effectiveness. Studies from Brazil and Chile have reported waning immunity with CoronaVac, leading to an increased incidence of breakthrough infections and prompting booster dose recommendations^9,19^. Given the observed low levels of Nab V2, our data suggest that booster doses tailored to emerging ­variants may be necessary to sustain neutralizing immunity, particularly against immune-evasive strains, although additional research is required to establish optimal vaccine strategies. Notably, ­heterologous booster strategies, such as ­administering an mRNA vaccine after an inactivated virus vaccine, have shown significantly improved neutralizing antibody responses compared to homologous ­boosting^23^. This supports the implementation of mixed vaccine regimens to enhance protection against ­emerging SARS-CoV-2 variants.

Recent studies have reinforced the importance of ­adapting booster strategies to emerging SARS-CoV-2 variants. Zhan et al. demonstrated that serum collected after XBB.1.5 booster vaccination displayed robust neutralizing activity against a broad range of variants^24^. Furthermore, a study published in 2025 indicated that, despite the natural decline in neutralizing antibody titers over time, vaccine efficacy against severe COVID-19 remained above 75%, highlighting that protection extends beyond measurable antibody levels alone^25^. These findings support the need for updated or heterologous booster formulations to improve cross-variant coverage and sustain long-term protection.

Overall, these findings emphasize the need for continuous monitoring of immune responses post-vaccination. Further longitudinal studies with larger cohorts and extended follow-ups are required to assess the long-term persistence of neutralizing antibodies and their role in preventing breakthrough infections. Additionally, future research should explore alternative vaccine strategies, including heterologous boosting and updated formulations targeting prevalent variants, to enhance the durability and breadth of immune protection.

This study has some limitations. The small sample size and short follow-up period limit the generalizability and long-term interpretation of the findings. Both assays used were qualitative, which precludes the quantification of antibody levels and subtle variations over time. Additionally, the inclusion of only healthcare professionals may introduce selection bias, and cellular immunity was not assessed, preventing a broader ­understanding of immune protection. These limitations should be considered when interpreting the findings and reinforce the need for multicenter studies with larger and more diverse cohorts to validate and expand upon these results.

## CONCLUSION

This study highlights the immune response dynamics following CoronaVac vaccination. IgG seroconversion peaked at D30, followed by stabilization. However, neutralizing antibody responses varied, with Nab V1 levels remaining stable and Nab V2 consistently low. While CoronaVac induces a humoral response, its neutralizing capacity against emerging variants may be limited.

Given the reduced neutralization capacity against variants of concern, booster doses may be essential for maintaining protection. Future studies should evaluate the long-term durability of neutralizing antibodies and strategies to optimize vaccine effectiveness against evolving SARS-CoV-2 variants.

## Data Availability

The datasets generated and/or analyzed during the current study are available from the corresponding author upon reasonable request.
